# Amyloid β Peptide Modifies Membrane Architecture and Surface Electrostatic Properties of Human Red Blood Cells

**DOI:** 10.3390/ijms262311361

**Published:** 2025-11-24

**Authors:** Galya Staneva, Vesela Yordanova, Avgustina Danailova, Ana-Maria Marinovska, András Dér, Stefka G. Taneva

**Affiliations:** 1 Institute of Biophysics and Biomedical Engineering, Bulgarian Academy of Sciences, “Acad. G. Bonchev” Str., Block 21, 1113 Sofia, Bulgaria; gstaneva@bio21.bas.bg (G.S.); vyordanova@biophys.bas.bg (V.Y.); avgustina_danailova@abv.bg (A.D.); anamariya.walter@gmail.com (A.-M.M.); 2 Department of Endocrinology, Faculty of Medicine, Medical University—Sofia, “Acad. Ivan Geshov” Blvd. No. 15, 1000 Sofia, Bulgaria; 3 Institute of Biophysics, HUN-REN Biological Research Centre, H-6701 Szeged, Hungary; derandra123@gmail.com

**Keywords:** red blood cells, amyloid beta, membrane lipid order, ζ-potential, confocal microscopy, Alzheimer’s disease

## Abstract

Abnormal accumulation of amyloid-beta (Aβ) peptides in the brain is a hallmark of Alzheimer’s disease (AD). Importantly, the peripheral blood cells are also exposed to the effects of pathological peptides that accumulate in AD. Herein, the interaction of Aβ42 oligomers (Aβ42) with human red blood cells (RBCs) and erythrocyte ghosts as in vitro models for AD is studied combining fluorescence spectroscopy, fluorescence microscopy, and electrokinetics. The binding of Aβ42 to RBCs was evidenced by the use of a fluorescent-labeled peptide. The membrane lipid order increased with the increase in both the Aβ42 concentration and the incubation time, creating a lipid–protein microenvironment characterized by higher molecular order and reduced heterogeneity in RBC membranes compared to control conditions. Notably, the increase in lipid order was less pronounced in erythrocyte ghosts than in intact RBCs. Furthermore, the ζ-potential measurements revealed Aβ42 induced alteration of the surface potential of RBCs in a concentration- and time-dependent manner, with freshly isolated RBCs exhibiting a highly negative potential that became increasingly negative at higher Aβ42 concentrations. These findings suggest that Aβ42 not only impacts neuronal function but also significantly alters the physical properties of RBCs that might compromise their function, potentially contributing to the systemic effects observed in AD.

## 1. Introduction

Neurodegenerative diseases are characterized by abnormal accumulation and misfolding of specific proteins, such as amyloid-beta (Aβ) peptide, tau (τ) protein, and α-synuclein (α-syn), in the brain and in peripheral tissues. Alzheimer’s disease (AD) is particularly associated with progressive accumulation of extracellular amyloid plaques of Aβ peptide (40 (Aβ40) to 42 (Aβ42) amino acids long) in the human brain [1,2,3,4,5,6,7,8,9,10], characterized by extracellular deposition of amyloid β (Aβ) peptides, predominantly Aβ40 and Aβ42, and intracellular neurofibrillary tangles composed of hyperphosphorylated τ-protein [11,12,13,14,15,16] that can cross the blood–brain barrier and reach the blood plasma [5,17]. Hence, the increased Aβ production in the brain may also be associated with higher Aβ concentrations in the blood of patients than in healthy subjects [18,19,20]. It should be noted here that the production of Aβ in healthy individuals represents a risk of AD development [21]. Thus, Aβ can affect not only neurons but also peripheral body fluids (plasma) and peripheral blood cells (erythrocytes (red blood cells, RBCs), platelets, and leukocytes) [22,23,24,25]. The Aβ peptide can be sequestered by a number of plasma proteins (albumin, fibrinogen, α2-macroglobulin, α1-antichymotrypsin, complement proteins, apolipoproteins, etc.) as well as by blood cells [22,23,24,25,26,27,28,29].

Different hypotheses have been proposed for the origin of AD. The most popular “amyloid cascade hypothesis” suggests a major role of amyloid plaques in AD [30,31,32,33,34,35,36] and is further modified to the “oligomeric amyloid hypothesis” that considers soluble Aβ oligomers as a trigger of the AD development [34,37,38,39,40,41,42]. Importantly, Aβ accumulation is detected not only in the brain of adults but also in young individuals [37,39,43], in patients with other pathologies [1,44,45,46,47,48,49] or brain injury [50,51], as well as in individuals without health problems [37,47,52,53]. All these studies suggest more complex pathophysiological mechanisms of AD [5,54]. Evidence for the unspecificity of Aβ plaques and soluble Aβ oligomers that contradict the “amyloid cascade hypothesis” are discussed in detail in [55]. On the other hand, generation of reactive oxygen species by Aβ that could induce oxidative damage of RBC membrane and in turn reduction of oxygen delivery from RBCs to the brain tissue has been reported [23,56]. The impaired oxygen release from RBCs to the brain and the related abnormal cellular metabolism have led to the erythrocytic hypothesis of the development of AD [55,57,58].

In addition, the involvement of intracellular Aβ peptides in mitochondrial dysfunction is the basis for the “mitochondrial cascade hypothesis” that considers the accumulation of individual mitochondrial dysfunctions along the process of cell aging, which affects Aβ peptide homeostasis and aggregation [59,60]. Nevertheless, there is uncertainty whether Aβ interaction with mitochondrial proteins and accumulation in the mitochondrial matrix contribute to mitochondrial disfunction in early stages of the disease or whether multiple pathological events can cause mitochondrial disorders [61,62,63]. Important information for this pathology is gained by in vitro studies of the Aβ interaction with membrane proteins [64], lipids [65,66], model membranes [67,68], and cells, including RBCs [69,70,71,72,73]. Molecular dynamics (MD) simulations and time-lapse atomic force microscopy (AFM) imaging revealed aggregation of Aβ42 on bilayer surfaces and dramatic transformation of Aβ42 monomers to misfolded, aggregation-prone conformations [74]. The conformational changes in Aβ are assumed to be strongly related to the membrane lipid composition [75,76]. Details of the initial stages of Aβ monomer–membrane interaction are provided by investigation on model lipid monolayers and bilayers containing negatively charged phospholipids in both the liquid-disordered (L_d_) and the liquid-ordered (L_o_) state [67,77,78]. The interaction is shown to be weak in the absence and stronger in the presence of negatively charged phospholipids (phosphatidic acid or cardiolipin) using a number of techniques and confirmed by MD simulations. MD has also shown that fibril formation on the membrane surface depends on the bilayer composition [77]. The binding of Aβ42 monomers to gangliosides containing sphingomyelin/cholesterol bilayers is shown to facilitate the aggregation of Aβ42, giving rise to more extended β-sheets [78]. Hence, lipid rafts, i.e., membrane domains enriched in sphingomyelin (SM) and cholesterol (Chol), are proposed to play a key role in the Aβ peptide aggregation at neuronal membranes and formation of toxic oligomers, and the high Chol content of cellular membrane lipid rafts favor the amyloidogenic pathway [68,79,80,81].

Furthermore, AFM-based imaging of blood reveals alterations in RBC size, shape, and morphology related to neurocognitive disorder and presence of fibrils assembled in crystals on the surface of RBCs from older AD patients [71]. The Girasole research group [82] demonstrates Aβ-induced changes in the membrane cytoskeleton and the membrane integrity, and accelerated morphological alterations and aging of human RBCs. In line with these studies, we have recently reported similar alterations in the morphology and nanomechanics of RBCs derived from patients with AD [83]. Using flow cytometry, Jayakumar et al. [69] reported that Aβ40 amyloid fibrils have high binding affinity for RBCs. The alterations in the RBC membrane architecture in AD subjects have been attributed to Aβ interactions with RBCs and/or to changes in the RBC membrane proteome [84].

In this work, we report on the interaction of Aβ42 oligomers (Aβ42) with freshly isolated human RBCs and hemoglobin-free human erythrocytes, so-called erythrocyte ghosts (RBC ghosts) as in vitro AD models, using a number of experimental approaches (fluorescence spectroscopy, fluorescence microscopy, and electrokinetics). Our findings demonstrate that Aβ42 interaction with both RBCs and erythrocyte ghosts is associated with changes in the membrane lipid order and surface charge. Fluorescence microscopy shows localization of Aβ42 on the cell surface.

## 2. Results

### 2.1. RBC Aging Monitored by Laurdan GP

The effect of Aβ42 on RBCs was studied at different oligomer concentrations (1–60 µM) and different incubation time (from 1 to 48 h). Peripheral plasma Aβ levels are typically in the pico- to nanomolar range [85,86]. The lowest concentration used here (1 µM) therefore seems to be supraphysiological for bulk blood but serves as a mechanistic probe of membrane interactions and may approximate local effective concentrations at microvascular interfaces or on RBC-bound aggregates. However, the bulk concentration is not the most relevant parameter for our experimental context. What is physiologically important is the effective ratio between red blood cell membrane lipids and Aβ molecules. At 20% hematocrit (Hct), the total concentration of RBC membrane lipids is approximately 2 mM, meaning that in our experiments, there are roughly 1000–2000 lipid molecules per amyloid molecule. This ratio does not represent an excessive Aβ load and supports the use of micromolar Aβ concentrations as mechanistic probes of lipid–protein interactions.

We first performed experiments with untreated RBCs at the same incubation time as for Aβ42-treated cells.

**Figure 1:**
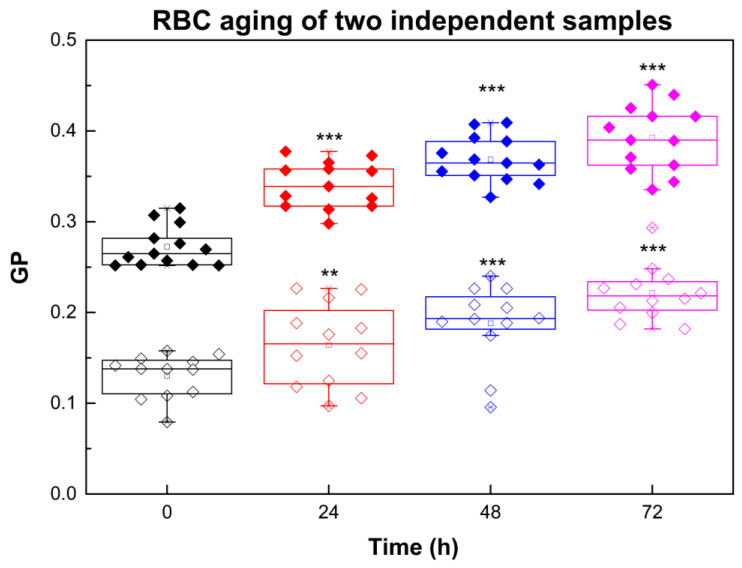
Laurdan GP of RBCs taken from two independent donors (sample 1 (filled symbols) and sample 2 (empty symbols)) as a function of time: black, 0 h; red, 24 h; blue, 48 h; purple, 72 h. For measurements of Laurdan fluorescence RBC suspensions with 20% Hct were diluted 200 times. Error bars correspond to the standard deviations (SDs). SD of 12 measurements per sample with 0.1% Hct at 37 °C at each studied time is shown. Nonparametric Mann–Whitney test was applied to determine *p* between control (0 h) and 24, 48 and 72 h: *p* < 0.01 (**); *p* < 0.001 (***).

The dataset in Figure 1 illustrates the typical time-dependent changes in Laurdan generalized polarization (GP) values that occur during RBC aging. Only two samples were presented in the RBC aging graphs, but all nine studied samples were analyzed both for aging alone and for aging in the presence of Aβ42. The graph demonstrates the direction and magnitude of the aging effect in terms of membrane lipid order, rather than to perform statistical analysis of aging across different samples. The statistics shown in the figure reflect the significance of GP differences between fresh and aged RBCs for each individual sample.

Aging of RBCs is related to GP increase (Figure 1) that corresponds to an increase in membrane dehydration/membrane lipid order.

As shown in Table 1, the temporal changes in GP values vary between individuals (data presented for two representative volunteers), with the most significant changes occurring within the first 24 h. Additionally, the rate of RBC aging was greater in membranes with lower initial lipid order (e.g., sample 2) compared to those with higher initial lipid order (e.g., sample 1). In other words, the more ordered the membrane at baseline, the smaller the relative GP increase over time. Although Figure 1 and Table 1 present data from two representative donors, the same inverse relationship between initial lipid order and the magnitude of the GP change was consistently observed across all nine RBC samples analyzed.

### 2.2. Interaction of Aβ42 Oligomers with RBC

**Figure 2:**
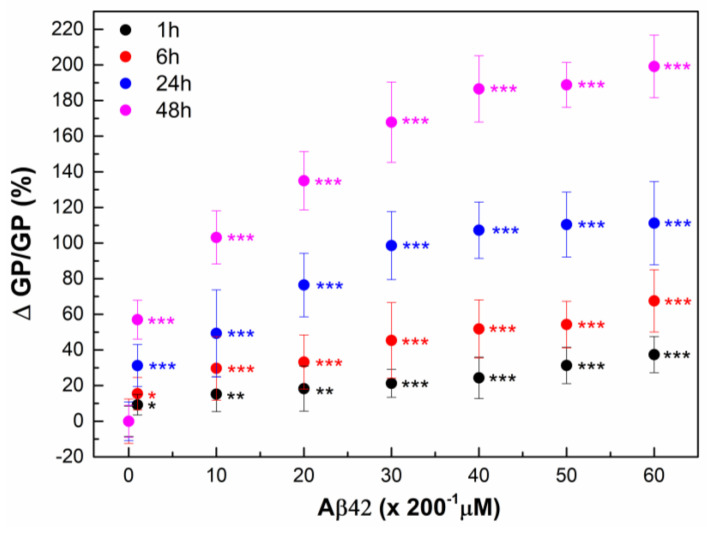
The relative change in Laurdan GP, ΔGP/GP (%), of RBCs was calculated as (((GP_Ci_ − GP_0_)/GP_0_) × 100) of 9 independent samples (individuals) as a function of time. RBC suspensions with 20% Hct were incubated with Aβ42 oligomers in concentrations from 1 to 60 μM (C_i_ = 1, 10, 20, 30, 40, 50 and 60 µM) for 1 (black)-, 6 (red)-, 24 (dark blue)- and 48 (pink)-hours (t_i_ = 1, 6, 24 and 48 h) at 37 °C. GP_0_ indicates GP value of non-treated RBCs (0 µM Aβ42). For measurements of Laurdan fluorescence the non-treated and Aβ42-treated RBC suspensions were diluted 200 times. The data represented 6 measurements of each RBC suspension with 0.1% Hct at 37 °C. SD of 54 measurements per point is shown. Nonparametric Mann–Whitney test was applied to determine *p* between control non-treated and Aβ42-treated RBCs: *p* < 0.05 (*); <0.01 (**); <0.001 (***).

**Figure 3:**
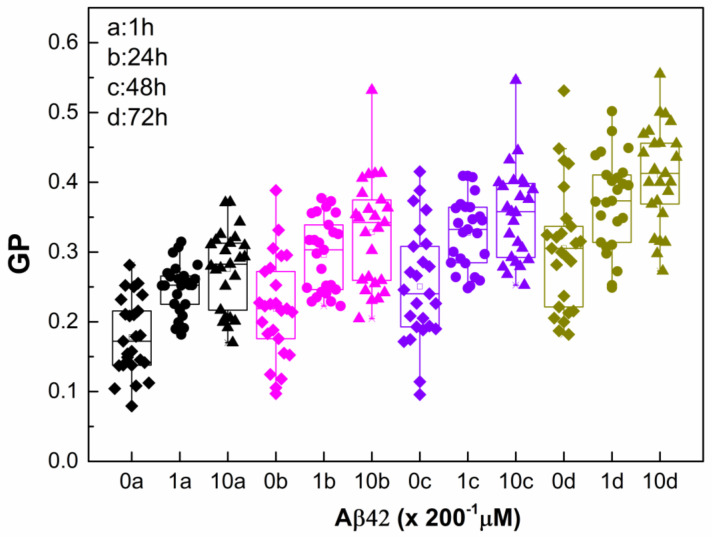
Laurdan GP of non-treated and Aβ42-treated RBCs taken from 2 independent samples as a function of time. The RBC suspensions with 20% Hct were treated with Aβ42 oligomers (0 µM (diamonds), 1 µM (circles), and 10 µM (triangles)), incubated for 1 h, washed and measured immediately (1 h (black)), and after 24 h incubation with Aβ42 oligomers at 37 °C, the samples were washed and measured at 24 (pink), 48 (purple), and 72 h (olive). For measurements of Laurdan fluorescence, the non-treated and Aβ42-treated RBC suspensions were diluted 200 times. SD of 12 measurements per sample with 0.1% Hct at 37 °C at each studied time is shown. Nonparametric Mann–Whitney test is applied to determine *p* between control non-treated and Aβ42-treated RBCs. Each studied concentration is significantly different from its control with *p* < 0.001.

Likewise, a noticeable increase in GP is observed with the increase in both the Aβ42 concentration and the duration of RBC incubation with Aβ42 (Figure 2 and Figure 3). A 1 h incubation results in a 10% increase in membrane lipid order at 1 µM Aβ42, a 20% increase at 5–40 µM Aβ42, and a 30% increase at 50–60 µM Aβ42. 30% increase in the membrane lipid order can also be achieved for 24 h incubation of RBCs with 1 µM Aβ42. Further increase in the membrane lipid order to 60% is reached for 48 h incubation time.

### 2.3. Monitoring RBC Aging with and Without Aβ42 Using GP

The data presented in Figure 3 highlight two parallel processes influencing membrane lipid order: the concentration-dependent effect of Aβ42 oligomers and the gradual aging of RBCs, both of which manifested as an increase in GP. The pronounced changes observed within the first day indicate that the structural perturbation of the RBC membrane is driven mainly by the direct interaction of Aβ42 with the membrane. Once the non-bound peptide is removed, ΔGP remains stable, suggesting that Aβ42 binding induces a relatively fixed alteration in membrane organization rather than a progressive effect. In addition, the continued increase in GP over time reflects intrinsic RBC aging process. Consequently, while the absolute GP values rise with time, the relative contribution of Aβ42 (ΔGP/GP) diminishes as RBCs age (Table 2). Both Aβ42 interaction and cellular aging promote higher membrane lipid order; however, the extent of this ordering differs. Aging of intact RBCs over 72 h leads to an approximately 70% increase in membrane lipid order, whereas Aβ42 alone induces about a 50% increase at the higher concentration, and aging of Aβ42-treated RBCs results in an approximately 120% increase. These findings suggest that Aβ42 binding modifies the membrane structure in a way that accelerates the aging trajectory in terms of lipid order, consistent with AFM data from Girasole’s group [87], showing that Aβ42 significantly accelerates morphological and nanomechanical alterations relative to normal aging.

### 2.4. Visualization of Aβ42-RBCs Interaction

**Figure 4:**
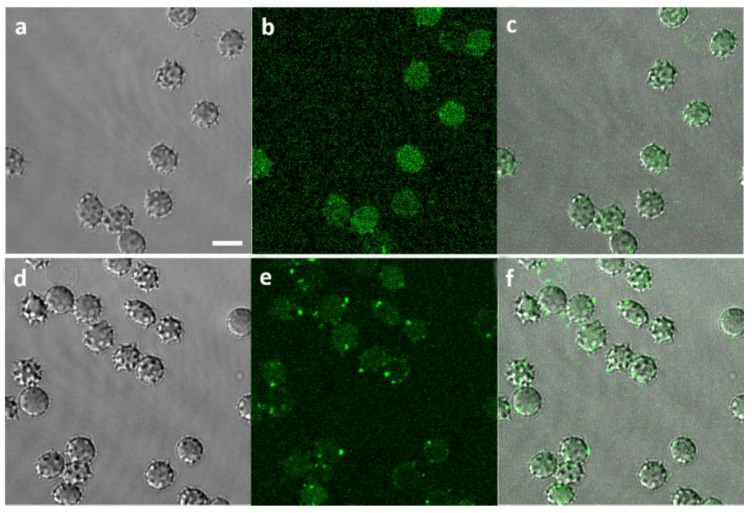
Visualization of Aβ42 binding to RBCs: (**a**) Phase contrast of RBCs incubated with 1 µM fluorescent Aβ (1–42) HiLyteTM Fluor 488 (Aβ*) for 48 h; (**b**) Green channel visualizing 1 µM Aβ42* binding to RBCs; (**c**) Merged channels at 1 µM Aβ42*/RBCs; (**d**) Phase contrast of RBCs incubated with 10 µM Aβ42* for 48 h; (**e**) Green channel visualizing 10 µM Aβ42* binding to RBCs; (**f**) Merged channels at 10 µM Aβ42*/RBCs. Scale bar: 5 µm.

Visualization of fluorescent Aβ42 proves Aβ binding to RBCs; higher Aβ42 concentration leads to the formation of larger Aβ42 aggregates on the RBC membranes (Figure 4). Some of these aggregates appear to be associated with the formation of RBC membrane spikes.

### 2.5. Plasma Membrane Lipid Order Visualization by Di-4-ANEPPDHQ Fluorescence Microscopy

**Figure 5:**
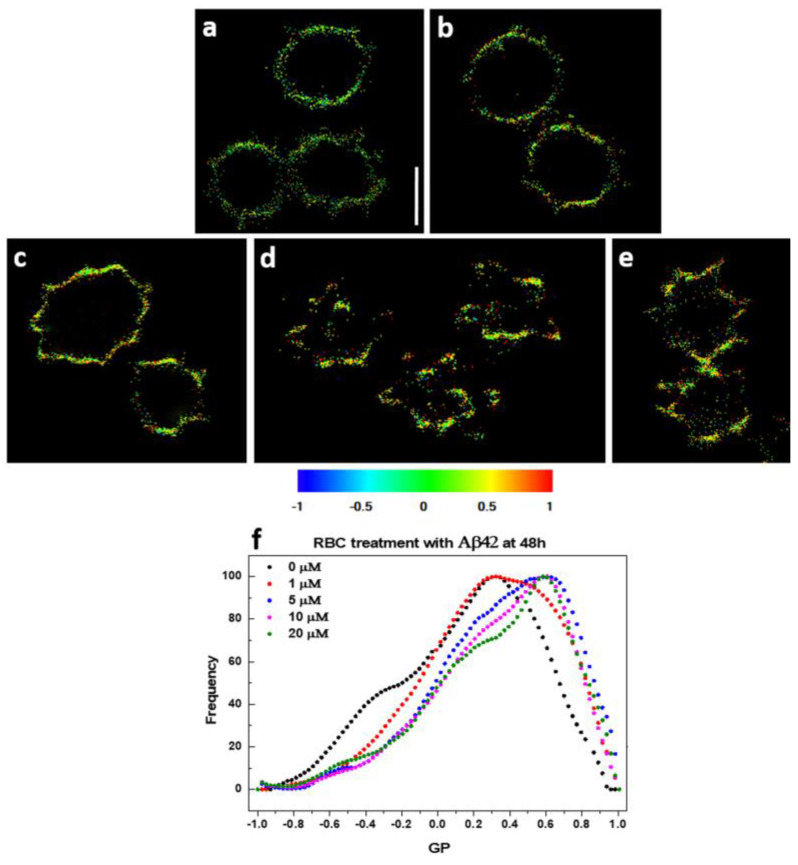
Calculated GP micrographs of control and Aβ42-treated RBCs stained with Di-4-ANEPPDHQ. (**a**) Untreated RBCs and RBCs treated with (**b**) 1 µM, (**c**) 5 µM, (**d**) 10 µM, and (**e**) 20 µM Aβ42 oligomers. Regions of high (red, GP = 1) and low (blue, GP = −1) membrane lipid order are visualized. Scale bar = 5 μm. (**f**) GP profiles of control and Aβ42-treated RBCs. Black curve corresponds to untreated RBCs; whereas red (1 µM), blue (5 µM), pink (10 µM) and green (20 µM) represent Aβ42-treated RBCs.

GP profiles of intact not-treated RBCs and cells treated with Aβ42 oligomers were monitored after 48 h incubation. The GP profiles shift to higher GP values in Aβ42 concentration-dependent way (Figure 5), and the higher the GP value, the higher the membrane lipid order.

**Figure 6:**
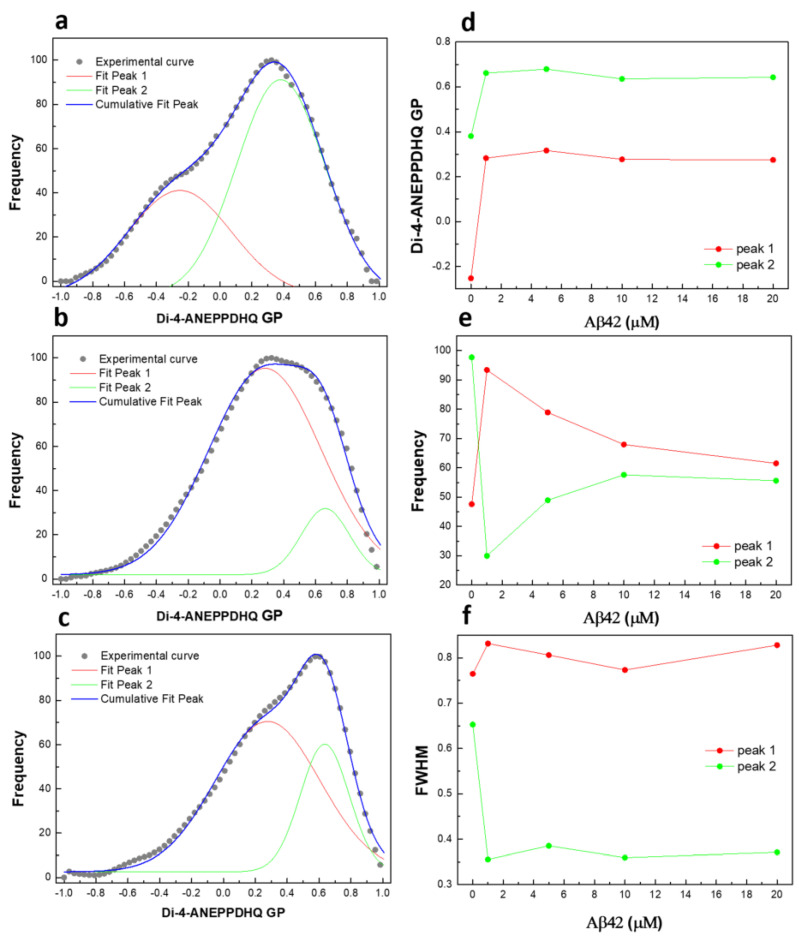
Deconvolution of GP profiles by two peaks: (**a**) untreated RBCs and cells treated with (**b**) 1 µM and (**c**) 10 µM Aβ42 oligomers at 48 h; (**d**) GP value at max frequency of each peak as a function of Aβ42 concentration; (**e**) frequency as a function of Aβ42 concentration; (**f**) full width at half maximum (FWHM) of each peak as a function of Aβ42 concentration.

Figure 6 demonstrates that at least two distinct GP profiles (microenvironments) exist in control untreated RBCs.

One profile is centered at −0.25, corresponding to membranes in the liquid-disordered phase (L_d_), while the other is centered at 0.38, representing membranes in the liquid-ordered phase (L_o_). Incubation of RBCs with 1 µM Aβ42 oligomers for 48 h increases the GP values of both profiles and alters the relative ratio between the two coexisting GP distributions, as well as their full width at half maximum (FWHM). Increasing Aβ42 concentrations up to 20 µM do not significantly affect the membrane lipid order of the distributions but do shift the proportions between the coexisting microenvironments. The FWHM quantifies the heterogeneity of the two coexisting microenvironments, revealing a clear difference in heterogeneity between Aβ42-treated and untreated RBCs. A decrease in FWHM describes a reduction in heterogeneity. Therefore, Aβ42 appears to induce a microenvironment with higher lipid order and a substantially lower degree of heterogeneity compared to the control conditions.

### 2.6. Interaction of Aβ42 Oligomers with RBC Ghosts

We used hemoglobin-free erythrocytes (RBC ghosts) for two main reasons. The first is technical—to avoid spectral overlap caused by hemoglobin absorption and fluorescence, as well as the potential quenching effect of hemoglobin on Laurdan emission [88]. The second is mechanistic—to distinguish the effects of Aβ42 interaction with membrane components from possible interactions with intracellular hemoglobin [89,90]. In addition, this approach allowed us to specifically assess the interactions of Aβ42 oligomers with the erythrocyte membrane, independent of intracellular components.

**Figure 7:**
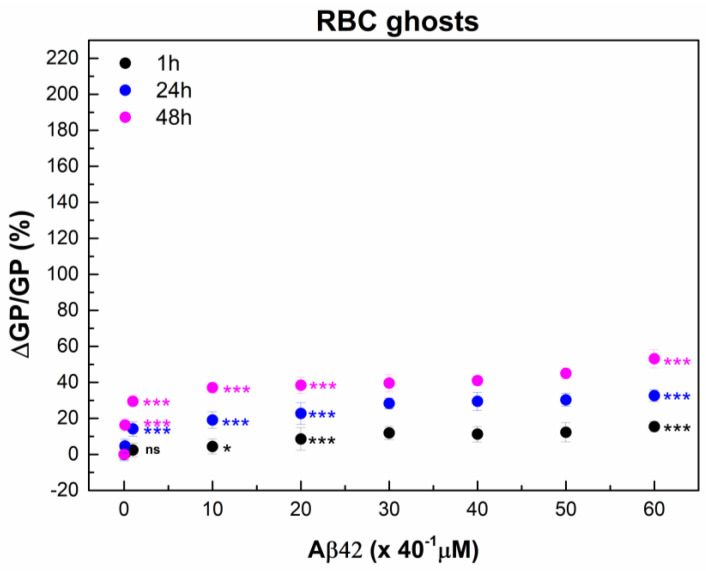
The relative change in Laurdan GP, ΔGP/GP (%), of RBC ghosts calculated as (((GP_Ci_ − GP_0_)/GP_0_) * 100) of 3 independent samples (individuals) as a function of time. RBC ghost suspensions with final total protein (TP) concentration of 2 mg/mL were incubated with Aβ42 oligomers in concentrations from 0.1 to 60 μM (C_i_ = 0.1, 1, 10, 20, 30, 40, 50, and 60 µM) for 1 (black), 24 (dark blue) and 48 (pink) hours (t_i_ = 1, 24 and 48 h) at 37 °C. GP_0_ indicates GP value of non-treated RBC ghosts (0 µM Aβ42). For Laurdan spectra measurements, non-treated and Aβ42-treated RBC ghost suspensions were diluted 40 times. The data represented 6 measurements of each RBC ghost suspension with 50 μg/mL TP at 37 °C. SD of 18 measurements per point is shown. Nonparametric Mann–Whitney test was applied to determine *p* between control non-treated and Aβ42-treated RBCs: *p* < 0.05 (*); <0.001 (***), and no statistically significant differences were denoted by ns.

Aβ42 induced a smaller increase in membrane lipid order in RBC ghosts compared to intact RBC membranes (Figure 2 and Figure 7). At incubation with 1 µM Aβ42, the change in membrane lipid order in RBC ghosts was two-fold lower than that observed in intact RBC membranes. Increasing Aβ42 concentration and incubation time progressively enhanced the difference in Aβ42-induced changes in membrane lipid order between RBC ghosts and intact RBC membranes.

### 2.7. ζ-Potential of Human RBCs After Interaction with Aβ42 Oligomers

**Figure 8:**
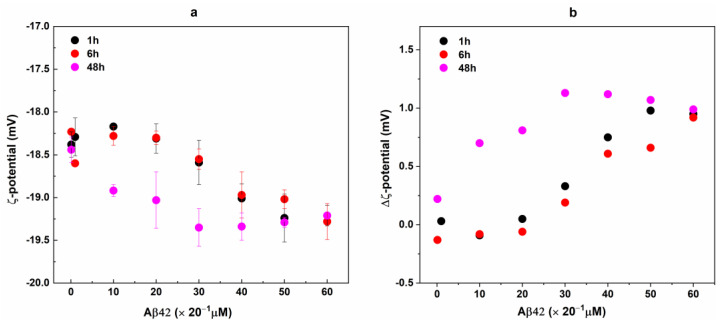
ζ-potential of Aβ42-treated RBCs taken from 3 independent samples each measured in 3 replicates (**a**). RBC suspensions with 20% Hct were incubated with Aβ42 oligomers in concentrations from 0.1 to 60 μM for 1 (black), 6 (red), and 48 (pink) hours at 37 °C. For ζ-potential measurements non-treated and Aβ42-treated RBC suspensions were diluted 20 times. The data represented 9 measurements of RBC suspensions with 1% Hct at 25 °C. Error bars corresponded to the standard deviations (SDs). The difference, Δζ, between the mean values of ζ-potential of non-treated (RBC_C = 0_) and Aβ42-treated RBCs (RBC_Ci_) at t = 1, 6, and 48 h (**b**).

To further validate the binding of Aβ42 to RBCs, we evaluate the ζ-potential of RBCs before and after interaction with Aβ42 oligomers. The ζ-potential, i.e., the electrostatic potential at the shear plane that separates the compact layer of strongly associated ions from the diffuse layer, is a measure of accessible surface charges on the cell surface [91,92]. ζ-potential measurements showed changes in the RBC potential in the presence of Aβ42 in a concentration- and incubation time-dependent manner (Figure 8a). The potential of fresh non-treated RBCs was −18.64 ± 0.27 mV; its absolute value became increasingly negative with increasing the Aβ42 concentration and was more negative for 48 h than for 1 and 6 h of RBC incubation with Aβ42. Importantly, significantly lower Aβ42 concentration of 20 μM induced the same change in ζ-potential at 48 h incubation time as 40 μM for 1 and 6 h of incubation of RBCs with Aβ42 (Figure 8a).

In Figure 8b, the difference between the ζ-potential of fresh non-treated RBCs (ζ_RBCt = 0_) (t = 0 h) and cells treated with Aβ oligomers (RBCs/Aβ42 complexes) (ζ_RBCci_) incubated for 1, 6 and 48 h (Δζ = ζ_RBCt = 0_ − ζ_RBCc = 0_ or ζ_RBCci_) is presented. The highest Δζ values of ca. 1 mV were estimated for the longest interaction time (48 h) at Aβ42 concentrations of 30 μM and above, while rather close values of Δζ were evaluated for 1 and 6 h incubation time. At the highest Aβ42 concentrations of 50–60 μM, Δζ reached a value of 1 mV after only 1 h incubation time.

**Figure 9:**
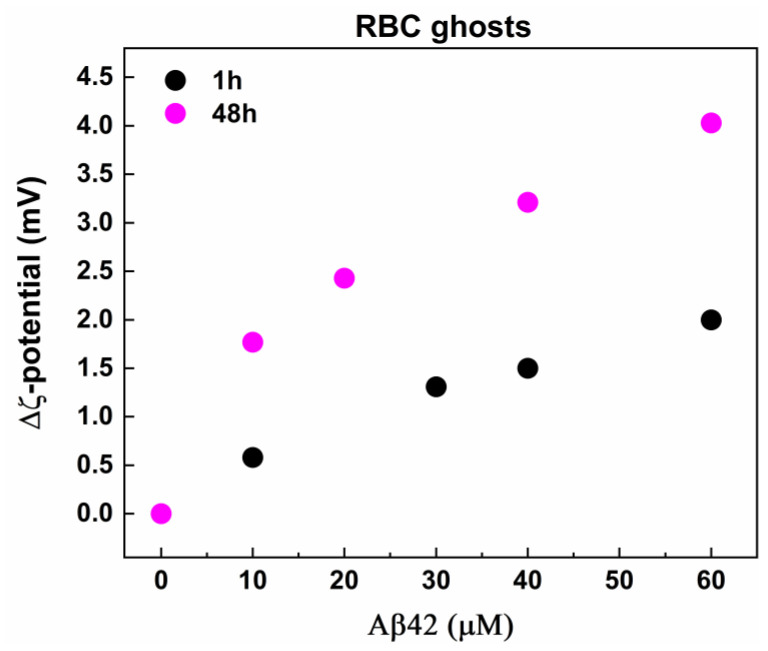
The difference, Δζ, between the mean value of ζ-potential of fresh non-treated RBC ghosts and the mean values of ζ-potential of non-treated and Aβ42-treated RBC ghosts with final TP concentration of 2 mg/mL incubated for 1 (black) and 48 (pink) hours at 37 °C. The ghost suspensions were treated with Aβ42 oligomers in concentrations from 0.1 to 60 μM and were measured without dilution of samples at 25 °C.

The ζ-potential of RBC ghosts was −33.28 ± 0.71 mV, a value significantly more negative than that determined for RBCs, which can be attributed to the different ionic strength of the buffers RBCs and ghosts were prepared. As with the case of RBCs, Aβ42 induced an increase in the negative ζ-potential of erythrocyte ghosts in a concentration-dependent way (Figure 9). The ζ-potential changes observed in Aβ42-treated ghosts were larger than those measured in Aβ42-treated intact RBCs at both incubation times (1 h and 48 h) (Figure 8b and Figure 9), suggesting a higher propensity of the ghosts to interact with Aβ42. This is likely due to the purification of the cell membranes, which removes not only hemoglobin but also other intracellular and membrane-associated components that could otherwise modulate the electrostatic interactions between the cells and Aβ42.

## 3. Discussion

Herein, we report on the interaction of Aβ42 oligomers, considered the most fibrillogenic and neurotoxic Aβ isoform, with RBCs and its effect on the cells’ physical features. The amphiphilic peptide is known to have a tendency to aggregate at hydrophilic–hydrophobic interfaces and at cell membrane surfaces [93,94,95], as well as affect RBC morphology and deformability [96]. Depending on the concentration, the peptide exhibits neurotrophic and neurotoxic properties [82,97].

Our data suggest that the RBC membrane architecture and surface properties are highly sensitive to the presence of Aβ42. We confirmed that Aβ42 binds to RBCs and forms large aggregates on the surface of the cell membrane. This is consistent with AFM data, which show development of crenatures, proto-spicules, and peculiar nanoscale features on the cell membrane upon Aβ42/RBC interaction, accompanied by acceleration of both the weakening of the cell cytoskeleton contacts and the age-related morphological and biochemical modifications of RBCs [82]. AFM imaging and computational modeling revealed aggregation of Aβ42 on the membrane surfaces and changes in the conformation of Aβ42 monomers [74]. In addition, AFM of smears of blood from patients with cognitive deficits display different types of protein aggregates (oligomers, protofibrils, and fibrils) on RBCs depending on patient age and the severity of the disorder, as well as crystals of fibrils for AD patients over 80 years old [71]. While previous RBC studies reported by Girasole et al. [82] emphasized nanotopography and mechanics (AFM morphology/roughness), our approach combines Laurdan GP (lipid packing) with ζ-potential (surface electrostatics) and uses hemoglobin-free ghosts to isolate membrane-intrinsic effects of Aβ42 oligomers, thereby providing complementary mechanistic insight.

Concomitant with formation of large Aβ42 aggregates on the cell membrane surface, we observed progressive increase in the membrane lipid order upon RBC interaction with higher Aβ42 concentration. It is worth mentioning that the fluorescence GP parameter shows that the lipid order also increases along the aging process. Moreover, the rate of cell aging depends on the membrane order, the less the membrane order the higher the aging rate. These results suggest an interaction of Aβ42 with RBC membrane lipids.

Binding of Aβ42 to membrane lipids has been studied extensively in model systems and damage to the bilayer structure has been reported [65,66,98]. It has also been found that as a consequence of the interaction with cell membrane lipids, the conformation of the peptide is changed [65,99] depending on the lipid composition [75,76,98]. It was suggested that the lipid rafts, e.g., nanodomains of cell membrane enriched in SM and Chol [67,100], are the place where oligomers and aggregates of exogenous Aβ42 are formed [80]. Furthermore, the interaction with SM is supposed to occur on the surface of cell membrane and that with Chol within the lipid bilayer, which might lead to fibrillization of the peptide and formation of aggregates, respectively [101]. A recent in vitro study has shown that gangliosides increased the Aβ42 peptide amount bound to the SM/Chol bilayer and facilitated peptide aggregation, but as the authors have discussed, the role of gangliosides at the nanoscale cannot be predicted [78].

Specialized submicrometric structures, enriched in Chol and sphingolipids, have been recently described in RBC membrane [102,103,104,105]. The RBC lipid bilayer, composed of phospholipids, a large proportion of Chol, as well as sphingolipids, is localized between the external glycocalyx layer and the membrane cytoskeleton, e.g., a network of proteins on the inner layer of the lipid bilayer [105]. The submicrometric domains are detected in the outer monolayer of RBC membrane, the Chol-enriched domains are preferentially linked to the edges, and the SM-enriched ones to the center of the membrane. Furthermore, the domains enriched in Chol are assembled at high curvature areas and those enriched in SM at low curvature areas of the membrane [101]. Using specific, sensitive, and quantitative probe (His-mCherry-theta-D4 (theta *)) capable of detecting Chol Carquin et al. [106] have shown clustering of lipids in two submicrometric domains at RBC membranes containing well-organized and less-organized Chol pools.

In concert with the above results, we found that at least two microenvironments exist in fresh RBC membranes (in the absence of Aβ42). This is consistent with X-ray diffraction data of oriented, multi-lamellar stacks of RBC membranes. The authors provide evidence of a patchy structure of RBC membranes composed of liquid-ordered (L_o_) and liquid-disordered (L_d_) lipid domains, as well as integral coiled-coil peptides domains [107].

Apparently, Aβ42 is able to change the lipid order of the two microenvironments and the proportion between the coexisting environments depending on the Aβ42 concentration. Thus, Aβ42 induced a microenvironment with higher lipid order and much lower degree of heterogeneity compared to untreated cells. Taking into account the earlier findings [101,106], we hypothesize that the peptide preferentially incorporates into the SM-enriched domains, contributing to the increase in the proportion of the ordered lipid phase. The glycosylated sphingolipids (e.g., GM1), together with the glycoproteins (e.g., Glycophorins A-D, or Band 3 protein) [108] form the hydrophilic glycocalyx layer [109], to which the hydrophilic N-terminus, which contains charged amino acid residues, is supposed to bind, while the hydrophobic C-terminus might anchor the bilayer. This is in line with the recent results of Yagi-Utsumi et al. [110] based on solid-state nuclear magnetic resonance studies, who have established that a unique antiparallel assemblage of Aβ42 is formed at the amphiphilic interface of the GM1-rich glycocalyx layer and the hydrophobic cell membrane, which then functions as a template for amyloid fibril formation.

We also found that the changes in RBC membrane architecture are accompanied by alteration of the cell surface electrochemical properties. The measured zeta potential, which reflects the surface electrical charge at the RBC membrane, increases with the increase in Aβ42 concentration and the time of treatment. Indeed, such an effect is expected when the peptide, possessing an overall negative charge, is associated with the membrane–glycocalyx interphase. Our results agree with the main conclusion of Chen et al. [111], according to which the decrease in the membrane elasticity results from the decrease in the surface charge of the cell. On the other hand, our results clearly demonstrate that an increased negative surface charge density can accompany a more rigid membrane structure in Aβ42-treated RBCs. Interestingly, the effect of Aβ42 on membrane lipid order was more pronounced in intact RBCs than in erythrocyte ghosts, whereas the ζ-potential changes showed the opposite trend—being larger in ghosts. This apparent discrepancy can be rationalized by considering the structural and compositional differences between intact RBCs and ghosts. In intact cells, Aβ42 interacts not only with the lipid bilayer but also with the glycocalyx, transmembrane proteins (such as Band 3), and the underlying spectrin–actin cytoskeleton [23,82,84]. The coupling between the bilayer and cytoskeleton allows Aβ42-induced changes in local lipid order to propagate and amplify throughout the membrane, leading to a measurable increase in overall membrane rigidity. In contrast, in ghosts, the absence of cytoplasmic components and partial loss of membrane-associated proteins reduce this cooperative coupling, so the same amount of Aβ42 produces smaller structural rearrangements in the lipid matrix. At the same time, ζ-potential reflects the net surface charge and the distribution of ions at the membrane–solution interface. The stronger ζ-potential changes observed in ghosts likely result from their exposed membrane surface and reduced shielding by extracellular proteins and glycocalyx components. In intact RBCs, the negatively charged sialic acid residues of the glycocalyx and adsorbed plasma proteins partially mask or buffer the electrostatic contribution of Aβ42 binding, whereas in ghosts, Aβ42 molecules can interact more directly with the phospholipid headgroups, enhancing the overall surface charge density. Thus, Aβ42 may affect different structural levels in intact RBCs (lipid–protein–cytoskeleton coupling) and in ghosts (lipid–electrostatic layer), explaining the opposing trends in lipid order and ζ-potential changes.

Beyond these membrane-related factors, intracellular components may also influence the electrostatic properties of intact RBCs. The ζ-potential of erythrocytes mainly arises from negatively charged sialylated glycoproteins, whereas intracellular iron is confined within hemoglobin and does not directly contribute to surface charge. Nevertheless, hemoglobin’s heme iron can participate in redox reactions that modify membrane components and local electrostatics. The amyloid β peptide family have been shown to bind hemoglobin through the heme moiety [89,90] and modulate iron redox states associated with oxidative stress [112]. Consequently, Aβ42 oligomers interacting with and partially internalizing into RBCs could affect hemoglobin’s redox balance, indirectly influencing the electrokinetic properties of intact cells. Although some residual hemoglobin remains in erythrocyte ghosts, as it has been detected by two photon excitation fluorescence microscopy [113], the presence of very low level of hemoglobin-bound iron and its associated redox effects may be related to the more negative ζ-potential.

These findings emphasize that Aβ42–membrane interactions are governed not only by lipid composition but also by the presence of protein and carbohydrate structures that modulate the mechanical and electrostatic properties of the cell. Therefore, intact RBCs represent a more physiologically relevant but also more complex system, where Aβ42 binding induces cooperative stiffening of the membrane, while the simpler ghost membranes reveal the direct electrostatic contribution of the peptide. Moreover, the use of RBC ghosts ensured that the observed alterations in membrane lipid order and electrostatic potential induced by Aβ oligomers reflected direct Aβ42–membrane interactions rather than any effects mediated by its interaction with intracellular hemoglobin.

Such membrane-level effects are particularly significant in the broader context of Alzheimer’s disease, where peripheral and neurovascular mechanisms are increasingly recognized. Beyond neuronal toxicity, RBCs can bind misfolded proteins, and RBC-associated aggregates have been proposed as disease-relevant readouts, with the 120-day RBC lifespan enabling temporal integration. Alterations in RBC membrane packing and surface electrostatics are expected to affect deformability and microvascular flow, with potential consequences for oxygen delivery and endothelial interactions in cerebral microvessels. Our findings therefore provide a biophysical link between Aβ42 oligomer exposure and RBC membrane properties relevant to neurovascular contributions in AD.

## 4. Materials and Methods

### 4.1. Preparation of RBC and Erythrocyte Ghost Suspensions and Treatment with Aβ42 Oligomers

Blood samples were derived from 10 healthy volunteers by venipuncture into EDTA (ethylenediaminetetraacetic acid) vacutainers (Becton, Dickinson and Company, Franklin Lakes, NJ, USA). The erythrocytes were isolated by centrifugation of the blood samples at 3000 rpm for 15 min at 4 °C and washed three times with PBS buffer (10 mM sodium phosphate, pH 7.2, 140 mM NaCl, and 1 mM EDTA) according to Girasole et al. [87].

To prepare erythrocyte ghosts, we followed the methodology described in [114]. Washed RBCs were hemolyzed in 5 mM Tris, pH 8.0 by centrifugation at 15,000 rpm and 4 °C for 30 min. This procedure was repeated until whitish ghost membranes were produced.

The hemoglobin (Hb) concentration of RBCs and total protein (TP) concentration of ghosts were determined spectrophotometrically (Specord 50 Plus, Analytik Jena, Jena, Germany). For TP concentration, the Biuret method was used [115]. RBCs with final Hb concentration of 67 mg/mL (20% hematocrit, Hct) and ghosts with final TP concentration of 2 mg/mL were incubated with the desired concentrations of Aβ42 in PBS and Tris buffer, respectively, at 37 °C for 1, 6, 24, 48 and 72 h under continuous gentle shaking. The unbound peptide was removed by centrifugation at 3000 rpm for 15 min and the suspensions were immediately used for the experiments after appropriate dilutions according to the requirements of the methods used, while maintaining the ratios of RBCs/Aβ42 and ghosts/Aβ42.

### 4.2. Preparation of Aβ42 Solutions

Aβ42 and fluorescent Aβ42 (HiLyte^TM^ Fluor 488) peptides were purchased from Eurogentec (Kaneka Eurogentec S.A., LIEGE Science Park, Seraing, Belgium). Nucleation of amyloid proteins leads to rapid aggregation and occurs quickly in a concentrated solution, so dissociating any preformed Aβ42 seeds is necessary to prevent aggregation. Briefly, to prepare monomeric aliquots, the lyophilized peptide was dissolved in pure HFIP (1,1,1,3,3,3-Hexafluoro-2-propanol > 99%, Sigma-Aldrich (St. Louis, MO, USA) to a concentration of 0.57 mg/mL, sonicated for 1 h, aliquoted, dried under argon, and held under vacuum for 2 h. The resulting dried Aβ42 aliquots were stored at −22 °C. Before each experiment, Aβ42 was dissolved in PBS followed by 2 min of vortexing. The Aβ42 solution was then filtered using a syringe filter with 0.45 µm and 0.22 µm pore size hydrophilic PVDF (polyvinyl difluoride) membranes (Millipore, Burlington, MA, USA; MILLEX-GV). No dimethyl sulfoxide (DMSO) was used for Aβ42 solubilization in our protocols. Aβ42 oligomers were prepared according to our previously published protocols [116,117]. Briefly, monomeric Aβ42 was dissolved in PBS (pH 7.4) to a concentration of 20–200 µM and incubated for 1 h at 37 °C, yielding predominantly oligomers, as verified previously by DLS, ThT fluorescence assays, and TEM. Depending on the desired final concentration of oligomers to be applied to RBCs or ghosts, aliquots of the oligomer stock solution were taken and added to the samples. Comparable 1 h oligomer formation in PBS has also been reported by other groups using independent biophysical techniques [118,119,120,121]. The physicochemical characterization of the oligomers has been published in [122]. The same holds for the preparation of fluorescent Aβ42 (Aβ42*) in PBS solutions. No mature fibrils were used in the present experiments. The choice of oligomers reflects their widely reported higher membrane activity and toxicity relative to monomers or fibrils [123].

For the imaging experiments, Aβ42 and (HiLyte™ Fluor 488)-labeled Aβ42 peptides were mixed in HFIP at 1:1 molar ratio.

### 4.3. Laurdan Fluorescence Spectroscopy of RBCs and Ghosts

The membrane lipid order was assessed by measuring the generalized polarization (GP) of Laurdan (a fluorescent probe localized in the membrane bilayer at the level of the phospholipid glycerol backbone). Laurdan GP provides information about the degree of hydration/lipid order in the polar head region near the glycerol backbone. Alterations in membrane water content cause changes in the excitation and emission spectra of the probe. Lower GP values (−1) correspond to a more disordered lipid membrane (L_d_) and higher values (+1) to a more ordered membrane (L_o_ or L_β_). Emission spectra were recorded from 390 to 600 nm. Samples were excited at 355 nm. GP is calculated using fixed emission wavelengths 440 nm and 490 nm (GP = (I_440_ − I_490_)/(I_440_ + I_490_)), where I_440_ and I_490_ are the emission intensities at a characteristic wavelength of ordered phase (440 nm) and of disordered phase (490 nm).

Laurdan, dissolved in DMSO in stock concentration of 0.25 mg/mL, was added to the RBCs (8 μL Laurdan) and ghosts (1 μL Laurdan) and incubated at 37 °C for 1 h. Measurements were performed by means of FP-8300 spectrofluorometer (Jasco Inc., Easton, MD, USA). The desired temperature (37 °C) was maintained using a circulating water bath (Julabo, Seelbach, Germany). For analysis of the steady-state spectra, OriginPro 9.0 software was used.

To verify that the observed changes in Laurdan GP values originated from direct interactions of Aβ42 oligomers with the erythrocyte membrane rather than from nonspecific effects of the surrounding medium, several control experiments were conducted. After incubation with Aβ42, RBCs were thoroughly washed to remove unbound oligomers. A GP shift was observed only when RBCs were incubated with Aβ42 oligomers, whereas the GP value of the corresponding supernatant was close to that of Aβ42 oligomers alone in PBS. Additional control experiments were performed in which Laurdan (dissolved in DMSO) was added to (i) buffer alone and (ii) Aβ42 oligomers dispersed in the same buffer. Both samples exhibited identical GP values, markedly different from those of RBC membranes. These results confirm that neither Aβ42 oligomers in solution nor components of the medium affect Laurdan fluorescence, indicating that the observed GP changes result from direct oligomer–membrane interactions.

### 4.4. Di-4-ANEPPDHQ Microscopy Measurements

Di-4-ANEPPDHQ staining was visualized using a laser scanning confocal microscopy (Carl Zeiss Microscopy GmbH, Jena, Germany) with a 100× immersion objective. GP calculations were based on the eq. GP = (I_500–580_ − GI_620–750_)/(I_500–580_ + GI_620–750_), as previously described [124]. Pseudo-colored GP images were merged with mean fluorescence intensity images to preserve structural information. We used a plugin to calculate GP and redeveloped for the CellTool software package by Georgi Danovski [125]. Thresholding of the images were performed before calculation of GP values in order to optimize the signal-to-noise ratio. The photomultiplier tube (PMT) gains were adjusted in such a way that both channels did not contain saturated pixels and have approximately the same intensity. As the assessment of the membrane order is a ratiometric measurement between the two channels, the PMT gain of each channel was kept constant between two sets of experiments, treated and non-treated with Aβ42 RBCs. The G factor for sensitivity between the two channels was calculated, and then GP values were assessed from raw fluorescent images and averaged from a minimum of 90 RBCs for each experiment performed at least three times. Data with *p* < 0.05 were considered significant when nonparametric Mann–Whitney test was applied.

### 4.5. Electrokinetic Measurements of RBCs and Ghosts

Measurements of ζ-potential were performed with Zetasizer (Nano zeta, Malvern Instruments, Malvern, UK) at 25 °C using disposable folded capillary cell cuvettes DTS1070 (Malvern, UK). The ζ-potential of RBCs and ghosts was determined in the absence and presence of Aβ42 at concentrations 0.1–60 μM, dissolved in buffer, after 1, 6, and 48 h of incubation at 37 °C. A minimum of three ζ-potential measurements were performed on each sample using the monomodal analysis model.

The electrophoretic mobility, u, was used for evaluation of the ζ-potential using the Smoluchowski equation [126]: ζ = 4πηu/ε, where η is the viscosity and ε is the dielectric permittivity of the solvent.

### 4.6. Statistical Analysis

All statistical calculations were performed with OriginPro 9.0 software. Data were analyzed with nonparametric Mann–Whitney test.

## 5. Conclusions

Our findings demonstrate that Aβ42 oligomers interact with RBC membranes in a concentration- and time-dependent manner, inducing pronounced alterations in both membrane structure and surface charge. The Aβ42 oligomers reorganize the lipid phase separation within RBC membranes and exert a stronger effect on membrane lipid order in intact cells than in erythrocyte ghosts. This difference reflects the cooperative roles of the spectrin–actin cytoskeleton and the glycocalyx in amplifying or modulating the peptide’s impact.

In parallel, Aβ42 enhances the negative ζ-potential of both RBCs and ghosts, with larger changes observed in the latter due to the absence of glycocalyx shielding and the more direct exposure of charged lipid headgroups. Together, these observations reveal that Aβ42 perturbs two interconnected levels of membrane organization: the lipid–protein–cytoskeleton continuum governing mechanical rigidity, and the electrostatic double layer controlling surface charge and ionic interactions.

Such dual modulation of RBC membrane properties may have functional consequences for blood rheology and oxygen transport, providing a plausible link between peripheral Aβ42 accumulation and microcirculatory impairment in Alzheimer’s disease. The present results support the emerging view that erythrocyte membranes are active participants in the systemic pathophysiology of neurodegeneration and may serve as accessible peripheral biomarkers for early detection of Aβ42-related disorders.

Future studies should aim to quantify the specific contributions of individual lipid species and cytoskeletal proteins to Aβ42 binding, as well as to explore how oxidative modifications or plasma components modulate these interactions under physiological and pathological conditions.

## Figures and Tables

**Table 1:** Relative change in GP (in % of GP for fresh RBCs, GP_0_) as a function of time calculated at t_i_ = 24, 48 and 72 h.

Sample	ΔGP at 24 h	ΔGP/GP at 24 h (%)	ΔGP at 48 h	ΔGP/GP at 48 h (%)	ΔGP at 72 h	ΔGP/GP at 72 h (%)
Sample 1	0.07 ± 0.03	25	0.09 ± 0.03	35	0.12 ± 0.03	44
Sample 2	0.03 ± 0.02	26	0.06 ± 0.05	44	0.09 ± 0.03	69

ΔGP/GP (%) = ((GP_ti_ − GP_0_)/GP_0_) × 100.

**Table 2:** Relative change in GP (%) of non-treated RBCs (GP_ti0μM_) and cells treated with Aβ42 oligomers (1 and 10 µM) for 24 h. The unbound Aβ42 was washed and ΔGP/GP was calculated for time t_i_ = 1, 24, 48, and 72 h.

Time (h)	Aβ42 (µM)	GP	ΔGP	ΔGP/GP (%)
1	0	0.1780		
	1	0.2479	0.0699	39
	10	0.2756	0.0976	55
24	0	0.2187		
	1	0.2961	0.0774	35
	10	0.3285	0.1098	50
48	0	0.2505		
	1	0.3276	0.0771	31
	10	0.3552	0.1047	42
72	0	0.3057		
	1	0.3714	0.0657	21
	10	0.4105	0.1048	34

ΔGP/GP (%) = ((GP_ti_ − GP_ti0μM_)/GP_ti0μM_) × 100.

## Data Availability

The raw data supporting the conclusions of this article will be made available upon obtaining the permission of the corresponding author for its use.
